# Unplanned reoperation after radical surgery for oral cancer: an analysis of risk factors and outcomes

**DOI:** 10.1186/s12903-022-02238-7

**Published:** 2022-05-25

**Authors:** Wei Zhang, Hong Zhu, Pu Ye, Meng Wu

**Affiliations:** 1 Department of Oral and Maxillofacial Surgery, The Affiliated Huaian No.1 People’s Hospital of Nanjing Medical University, Huaian, 223300 Jiangsu Province China; 2 Department of Pharmacy, The Affiliated Huaian No.1 People’s Hospital of Nanjing Medical University, Huaian, 223300 Jiangsu Province China

**Keywords:** Reoperation, Risk factors, Treatment outcome, Survival analysis

## Abstract

**Background:**

Unplanned reoperation (UR) after radical surgery for oral cancer (OC) is a health threat for the patients. The aim of the study was to identify the incidence of and risk factors for unplanned reoperation following oral cancer radical surgery, and to explore a potential role for long-term survival.

**Methods:**

The present study followed a retrospective study design. Univariate and multivariate analyses were used to identify risk factors for demographic and clinical characteristics of patients. Survival analysis was performed by the Kaplan–Meier method. The data was analyzed statistically between November and December 2021.

**Results:**

The incidence of UR was 15.7%. The primary cause of UR was reconstructed flap complications. Multivariate logistic regression analyses revealed that diabetes, tumor size, type of reconstruction, and nodal metastasis were independent risk factors for UR. Patients undergoing UR had a longer hospitalization, more post-operative complications, and a higher mortality compared with the non-UR group. UR is negatively correlated with the cancer-specific survival rate of patients (*Log-rank test, P* = *0.024*).

**Conclusion:**

Diabetes, tumor size, pedicled flap reconstruction and cervical nodal metastasis (N2) as independent risk factors for UR was discovered. UR was positively correlated with perioperative complications prolong hospital stay, and increased early mortality, but negatively correlated with the cancer-specific survival rate survival rate.

## Background

Oral cancer (OC) ranks as the sixth most common carcinoma, and it’s a matter of global concern [1]. The higher prevalence and mortality of oral cancer have been reported in developing countries, and China has been perceived as a high incidence of oral cancer [1, 2]. Rapid diagnosis can better control the transformation of precancerous lesions to oral cancer, and improve the overall survival rate of patients [3]. Along with technological developments, tissue fluorescence imaging and molecular biomarkers are used extensively in the cancer clinic [3, 4]. While these technologies have attractive advantages, they all have disadvantages. For example, the characteristics of false-positive, insufficient diagnostic ability, and the clinical diagnostic ability have not been recognized internationally. Therefore, histopathological examination currently remains the diagnostic gold standard. Surgery is considered to be the most effective modalities for the treatment of oral cancer patients [5]. Despite continuing improvements in surgical methods, reducing the complications and improving long-term survival remains a concern. Due to the intricacy of the surgery, postoperative 30-day complication rates for oral cancer can be as high as 20.3% [6]. Approximately 10% of patients with these complications require reoperation [6].

In the perioperative period, patients with surgical complications may undergo unplanned reoperation. Unplanned reoperations have been extensively researched in a variety of surgical fields [7–10]. Previous studies have established that unplanned reoperation or readmission may be associated with colorectal cancer and brain tumor recurrence and mortality [11, 12]. Unplanned reoperations are not expected by surgeons and patients, which will bring physical and psychological pressure to patients, increase hospitalization costs, and put pressure on social resources [13]. Unplanned reoperations account for between 2 and 10% of all surgeries in most surgical areas [14–17]. Several studies examined the risk factors of unplanned reoperation for head and neck cancer surgery complications. Choi et al. showed that long operation time, previous treatment, and higher N (N2) classification were considered key risk factors for unplanned reoperation [18]. According to Zhao et al.’s study, the leading cause of UR were postoperative bleeding, vascular crisis, and diagnostic issues, and patients with microvascular flaps or malignant tumors [19]. Up to now, far too little attention has been paid to assessing the incidence and risk factors associated with UR for oral cancer. To our knowledge, no previous study has given sufficient consideration to the impact of UR on long-term survival after radical oral cancer surgery. Thus, there is a great need to understand the relationship between UR and cancer prognosis.

The aim of the study was to identify the incidence of and risk factors for unplanned reoperation following oral cancer radical surgery, and to explore a potential role for long-term survival. The result from the study may increase the attention of UR among clinicians and reduce the incidences of postoperative complications after surgery.

## Methods

### Materials and methods

**Fig. 1:**
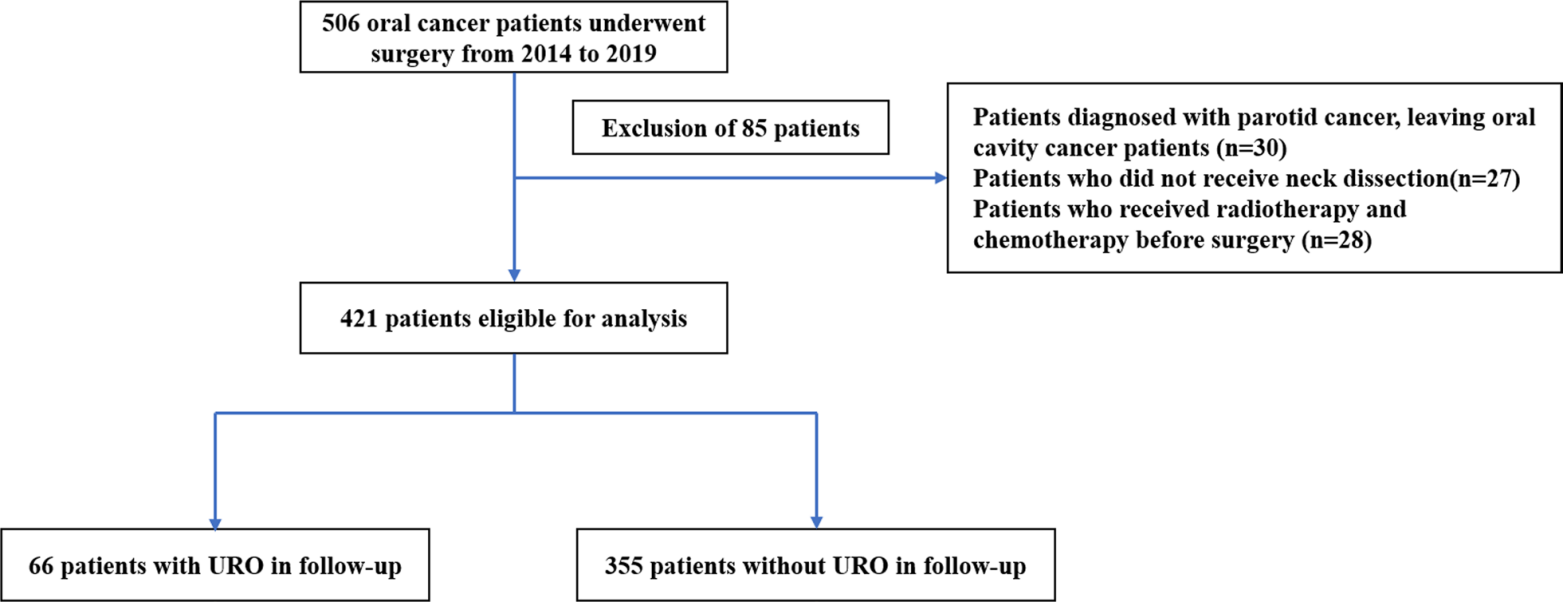
Flowchart of patient selection. UR, unplanned reoperation

A retrospective analysis was done on oral cancer patients treated with radical oral cancer surgery in the Department of Oral and Maxillofacial Surgery, The Affiliated Huaian No.1 People’s Hospital of Nanjing Medical University. 506 patients undergoing radical surgery for OC between February 2014 and November 2019 were included. The exclusion criteria were as follows: (1) maxillofacial salivary cancer patients, leaving oral cavity cancer patients (n = 30); (2) patients who did not receive neck dissection (n = 27); (3) patients who received radiotherapy and chemotherapy before surgery (n = 28). Together, 421 patients were enrolled for further analysis. According to postoperative assessment, 66 patients were assigned to the UR group, and 355 patients were termed the non-UR group (Fig. 1). All patients were followed for up to 2 years. We strictly followed 1975 Declaration of Helsinki ethical guidelines. This study was approved by the ethical review board of The Affiliated Huaian No.1 People’s Hospital of Nanjing Medical University.

### Data collection and definitions

Basic information on patients, body mass index (BMI), medical history, surgical procedures, postoperative complications, and the annual follow-up data were derived from the medical record database of The Affiliated Huaian No.1 People’s Hospital of Nanjing Medical University. OC was diagnosed according to histopathological evaluation, and the histological grade was staged according to the AJCC-TNM staging [20]. UR refers to the status that the occurrence of re-operation and retreatment follow-up 30 days after surgery due to complications caused by oral cancer surgery. The cancer survival rate was defined as the time from the first day of treatment performed to death.

### Operative techniques

Experienced surgeons perform all operations in oral and maxillofacial surgery at our center. At least 50 radical surgeries for oral cancer have been performed by surgeons. The operation method was decided using a risk-adapted approach based on tumor location and size, according to the treatment principle for oral cancer [21, 22]. Free or pedicled flaps were adopted for large postoperative defects' reconstruction after surgery: submental island flap, forearm flap, fibula flap, and anterolateral thigh flap.

### Follow-up

A flow chart presenting patient selection is displayed in Fig. 1. Follow-up data were collected from all patients who survived the UR procedure (n = 66), and 355 patients in the non-UR group. Telephone follow-up, medical records, visiting, or outpatient/inpatient clinic visits were used to collect follow-up data. The deadline for follow-up was December 31, 2021.

### Statistical analysis

Univariable and multivariable analysis were used to evaluate clinical factors for unplanned reoperation. Categorical data were analyzed by Chi-square test or Fisher’s exact test. Continuous data were compared with the Mann–Whitney U test. A multivariate Cox regression analysis and Kaplan–Meier analysis were utilized to assess recurrence-free and overall survival of UR. All data analyses were performed with the SPSS (IBM SPSS 22.0, SPSS Inc). Statistical tests were two sided and considered significant with a *P*-value < 0.05.

## Results

### Clinical variables between UR group and non-UR group

421 patients were enrolled in the study, included 227 males and 194 females. The mean age was 65.0 ± 8.0 years, 66cases (mean age: 71.1 ± 10.1 years) underwent UR, including 41 males and 25 females. The incidence of UR was 15.7% (66/421). The leading cause of unplanned reoperation was reconstruction flap related complications (32/66, 48.5%), followed by bleeding (9/66, 13.6%), necrosis (7/66, 10.6%), infection (6/66, 9.1%), fistula (2/66, 3.0%), and flap donor site complications (2/66, 3.0%) (Table 1). Gender, age, smoking, drinking, BMI, preoperative anemia, tumor size and cervical node metastasis did not show a significant difference in UR group and non-UR group. Early tumor (T1-T2) and no lymph node metastases (N0) were the most common cancer types in the UR group (86.4% and 75.8%, respectively) and the non-UR group (88.2% and 83.4%, respectively) (Table 2).

**Table 1:** Causes of UR within 30 days in oral cancer surgery (n = 66)

Causes	No. (%)
Reconstructed flap complications	32(48.5)
Bleeding	9 (13.6)
Necrosis	7 (10.6)
Infection	6 (9.1)
Fistula	2 (3.0)
Flap donor site complications	2 (3.0)
Others	8 (12.2)

UR, unplanned reoperation

**Table 2:** Univariate analysis of potential influencing factors for UR

Variables	Non-UR	UR	*P*-value
	Group (n, %)	Group (n, %)	
Gender			0.1786
Male	186 (52.4)	41 (62.1)	
Female	169 (47.6)	25 (37.9)	
Age			0.7737
< 60	111 (31.3)	22 (33.3)	
≥ 60	244 (68.7)	44 (66.7)	
Smoking			0.6661
No	318 (89.6)	58 (87.9)	
Yes	37 (10.4)	8 (12.1)	
Alcohol			0.6232
No	328 (92.4)	60 (90.9)	
Yes	27 (7.6)	6 (9.1)	
BMI (kg/m^2^)			0.5823
≤ 25	215 (60.6)	43 (65.2)	
> 25	140 (39.4)	23 (34.8)	
Hypertension			0.0367*
No	262 (73.8)	40 (60.6)	
Yes	93 (26.2)	26 (39.4)	
Diabetes			0.0128*
No	318 (89.6)	51 (77.3)	
Yes	37 (10.4)	15 (22.7)	
Preoperative anemia			
No	292 (82.3)	54 (81.8)	> 0.9999
Yes	63 (17.7)	12 (18.2)	
Tumor size			0.6821
T1–T2	313	57	
T3–T4	42	9	
Cervical node metastasis			0.2457
N0	296 (83.4)	50 (75.8)	
N1	35 (9.6)	11 (16.7)	
N2	24(7.0)	5 (7.5)	
Type of reconstruction			0.0002*
Local flap	283 (79.7)	37 (56.1)	
Pedicled flap	38 (10.7)	17 (25.8)	
Free flap	34 (9.6)	12 (18.1)	
Cancer subsites			0.0390*
Oral cavity	304	61	
Oropharynx	9	4	
Larynx	7	0	
Salivary Gland	35	1	

^*^P < 0.05

UR, unplanned reoperation; BMI, body mass index

Furthermore, the multivariate analysis showed that diabetes (OR = 2.544, 95%CI 1.257–5.128, *P* = 0.009), tumor size (OR = 1.879, 95%CI 1.038–3.401, *P* = 0.037), N classification (N2, OR = 3.076, 95%CI 1.526–6.211, *P* = 0.002) and type of reconstruction (pedicled flap reconstruction, OR = 0.491, 95%CI 0.259–0.931, *P* = 0.029) were independent risk factors for unplanned reoperation in OC patients (Table 3).

**Table 3:** Multivariate analysis of potential influencing factors for UR

Variable	Multivariate survival analysis		
	Hazard ratio	95% CI	*p*-value
Gender	0.997	0.616–1.613	0.991
Smoking	1.180	0.424–3.286	0.751
Alcohol use	0.722	0.242–2.154	0.559
BMI (kg/m^2^)	1.282	0.751–2.189	0.362
Hypertension	1.519	0.792–2.914	0.208
Age	0.550	0.036–8.385	0.667
Diabetes	2.544	1.257–5.128	0.009*
Preoperative anemia	0.643	0.374–1.104	0.109
Tumor size	1.879	1.038–3.401	0.037*
Cervical nodal metastasis			
N0			
N1	2.277	0.918–5.649	0.076
N2	3.076	1.526–6.211	0.002*
Type of reconstruction			
Local flap	Ref		
Pedicled flap	0.491	0.259–0.931	0.029*
Free flap	0.399	0.151–1.055	0.064
Cancer subsites			
Oral cavity	Ref		
Oropharynx	0.591	0.059–5.927	0.655
Salivary gland	0.738	0.780–7.004	0.791

Numbers in boldface indicate statistically significant values (*P* < 0.05)

^*^P < 0.05

UR, unplanned reoperation; BMI, body mass index

### Early surgical outcomes of the UR patients

The incidence of postoperative complications after surgery were 77.3% (51/66) in UR group while the incidence for non-UR group was 6.5% (23/355), including reconstructed flap complications, infection, bleeding, fistula, flap donor site complication and necrosis. Additionally, lengths of stay in the UR group had a significantly increased compared with the non-UR group (23.78 ± 0.82 vs 13.24 ± 0.32 days, *P* < 0.001) (Table 4).

**Table 4:** Early surgical outcomes for patients with or without UR

Parameters	Non-UR group (n = 355)	UR group (n = 66)	*P*-value
Hospital stay (days)	13.24 ± 0.32	23.78 ± 0.82	< 0.001*
Postoperative complications	23(6.5%).^a^	51(77.3%).^b^	< 0.001*
Mortality rate	0(0.0%).^a^	2(3.0%).^b^	0.024*

*P < 0.05

^a^Parameters in the non-UR group refer to data after the initial operation

^b^Parameters in the UR group refer to data after the second operation

UR, unplanned reoperation

### UR as a prognostic factor for long-term survival

**Fig. 2:**
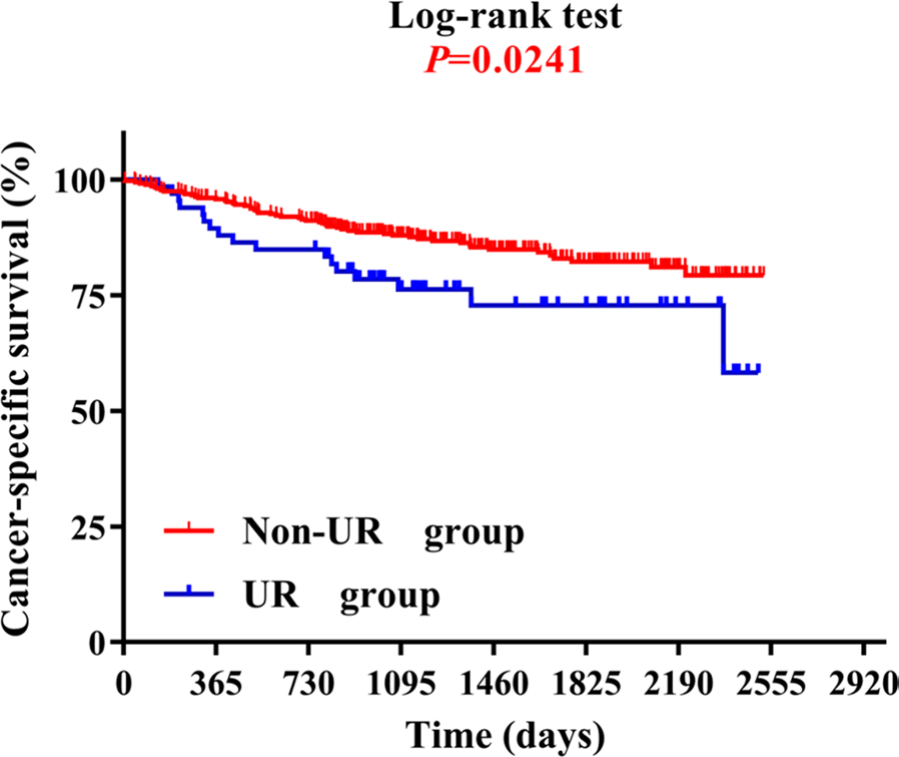
Kaplan–Meier curves for cancer-specific survival. UR, unplanned reoperation

In the survival analysis of OC patient prognosis, the mean follow-up time was 53 months. According to the univariate survival analyses, unplanned reoperation, type of reconstruction, age, diabetes, preoperative anemia, tumor size, and cervical nodal metastasis may be predictive factors for recurrence-free survival (Table 5). Furthermore, the results of multivariate logistic regression analysis showed that age (HR = 3.077, 95%CI 1.664–5.682, *P* < 0.01), diabetes (HR = 1.833, 95%CI 1.091–3.257, *P* = 0.02), N classification (N1, HR = 4.464, 95%CI 2.551–7.813, *P* < 0.01) (N2, HR = 2.315, 95%CI 1.101–4.878, *P* = 0.03), type of reconstruction (Pedicled flap, HR = 0.413, 95%CI 0.236–0.722, *P* = 0.02) (free flap, HR = 0.354, 95%CI 0.148–0.801, *P* = 0.01), and unplanned reoperation (HR = 2.864, 95%CI 1.181–7.401, *P* = 0.02) were independent predictors of outcome (Table 5). UR was significantly associated with shortened cancer-specific survival in this study (Log-rank test, *P* = 0.024) (Fig. 2).

**Table 5:** Prognostic factors for cancer-specific survival after curative oral cancer resection in univariate and multivariable analyses

Variable	Univariate survival analysis			Multivariate survival analysis		
	Hazard ratio	95% CI	*p*-value	Hazard ratio	95% CI	*p*-value
Gender	0.987	0.633–1.541	0.955			
Smoking	0.927	0.446–1.927	0.838			
Alcohol use	0.950	0.413–2.186	0.904			
BMI (kg/m^2^)	0.651	0.400–1.060	0.084			
Hypertension	1.263	0.780–2.047	0.342			
Age	2.312	1.297–4.123	0.005*	3.077	1.664–5.682	< 0.001*
Diabetes	2.693	1.583–4.582	< 0.001*	1.833	1.091–3.257	0.023*
Preoperative anemia	2.006	1.246–3.229	0.004*	0.678	0.415–1.109	0.121
Tumor size	2.074	1.257–3.421	0.004*	0.638	0.379–1.074	0.091
Cervical nodal metastasis						
N0	Ref					
N1	4.878	2.873–8.264	< 0.001*	4.464	2.551–7.813	< 0.001*
N2	2.667	1.300–5.464	0.007*	2.315	1.101–4.878	0.027*
Type of reconstruction						
Local flap	Ref					
Pedicled flap	0.433	0.257–0.729	0.002*	0.413	0.236–0.722	0.002*
Free flap	0.336	0.148–0.763	0.009*	0.345	0.148–0.801	0.013*
Cancer subsites						
Oral cavity	Ref					
Oropharynx	1.658	0.605–4.545	0.325			
Larynx	1.390	0.254–7.590	0.704			
Salivary Gland	1.041	0.116–9.350	0.971			
URO	2.768	1.118–6.854	0.028*	2.864	1.181–7.401	0.020*

Numbers in boldface indicate statistically significant values (*P* < 0.05)

^*^P < 0.05

UR, unplanned reoperation; BMI, body mass index

## Discussion

The incidence of oral cancer is increasing year by year, which has become a global concern [23]. The sooner OC is diagnosed, the better the therapeutic efficacy was detected. The fluorescence method is a non-invasive way of diagnosing oral cancer, but it is still in clinical exploration due to the occurrence of false positives [24]. Histopathological examination currently remains the diagnostic gold standard. At present, physicians and scholars are devoted to the study of oral prognosis, but it is affected by many factors [25]. In recent years, UR is garnering more and more attention.

Over the last dozen years, the incidence of UR has decreased with the advances in surgical technique. Nevertheless, the poor prognosis among patients with UR remains a serious threat to global health. UR often is associated with higher postoperative morbidity, mortality and consequent high medical costs [26, 27]. In the current medical and health institutions, the UR rates was recognized as one of the evaluation indicators of surgical quality, attracting the attention of clinicians [8]. According to Choi et al.’s study, the incidence of unplanned reoperation in head and neck cancer surgery was 10.5% [18]. Zhao et al. reported that the overall unplanned reoperation rate in oral and maxillofacial surgery was 1.52% [19]. The incidence of UR was found to be 15.7 percent in this study, which enrolled over 400 patients. UR is typically correlated with complex surgical procedures such as radical surgery of oral cancer. To further lower risk factors and indications for UR, it is meaningful for surgeons to understand preoperative, perioperative, and postoperative risk factors. Meanwhile, we found that diabetes, tumor size, N classification (N2), and pedicled flap reconstruction were independent risk factors for UR.

Flap failure was the most dominant cause of unplanned reoperations in our study. Thomas’s comparative study showed a reoperation rate of 20.2% in the head and neck free flap reconstructions [28]. Recently, Kwok and Agarwal had investigated the reoperation rate of all microvascular free tissue transfers, and showed the overall reoperation rate was 12.9% and a head and neck reoperation rate was 18.0% [29]. Blockage of blood arteries owing to thrombus development was discovered to be a major cause of flap failure [30]. Anastomosis of a secondary vein has been proven beneficial to the survival of the flap and reduces venous congestion [30]. However, microscopic vascular anastomoses required enormous amounts of time to expend. Moreover, multiple studies showed that a single venous anastomosis can provide enough drainage while maintaining flap survival and reducing operation time, with no significant difference compared to two venous anastomosis [31, 32]. To prevent venous thrombosis, Matti Sievert et al. suggested that low molecular weight heparin can effectively improve the survival rate of the flap [33]. The above literature showed that one venous anastomosis combined with low molecular weight heparin applied could be attributed to an increased survival rate of flap.

Previous studies have found that postoperative bleeding is the most common reason for unplanned reoperation for a variety of diseases, including maxillofacial surgery [17, 34, 35]. Our data revealed that postoperative bleeding was the second most common complication after radical oral cancer surgery. There were only 9 cases of postoperative hemorrhage in UR group, accounting for nearly 13.6 percent of the total number of UR patients. Early postoperative bleeding is associated with hemostasis failure or coagulation defects [19]. Taking NSAIDS and hypertension together is a common cause of postoperative bleeding [36, 37]. In our opinion, preoperative coagulopathy should be given particular attention, and blood pressure should be maintained normal in individuals. Moreover, controlling hemostasis and blood pressure under anesthesia is critical intraoperatively [38]. In this study, delayed hemorrhages were more common in tiny arteries and muscle or connective tissue in the surgical area. To prevent hemostasis, an electrical knife has been widely used in a variety of surgical specialties. Zhao et al. considered that the postoperative bleeding was caused by inappropriate use of the electrical knife [19]. Electrical knives with a high frequency should stay in the tissue for a long enough time to establish effective hemostasis. The ultrasonic scalpel can be effective for providing a relative bloodless field in muscle or connective tissue, which facilitates saving surgical time [39]. Persistent postoperative pain promotes high blood pressure, which also leads to arrhythmia or small blood vessel hemorrhage. Sufficient postoperative analgesia is more conducive to reducing the risk of UR.

Marra et al. found that extra-nodal extension and perineural invasion are prognostic factors associated with reduced disease-free survival for oral tongue cancer [40]. Hussain et al. showed that nodal stage was the most important poor prognostic factor in terms of disease-free survival in T1, T2 oral tongue cancer [41]. Our research showed that UR may have a significant impact on oral cancer patients' long-term survival rates following surgery. In addition, multivariate analyses revealed that unplanned reoperation may be poor prognostic factors for cancer-specific survival.

Despite our efforts to subject it to a comprehensive analysis, the present study still has a few limitations. First, our study was based on retrospective single-center study. Further prospective multicenter studies may be warranted to further validate the findings. Second, the relatively small sample size of UR should be taken into consideration. Third, due to the complex condition of patients, different clinical decision-making for the same disease might affect our results. Therefore, a more comprehensive investigation is needed for further confirmation in the context of big data. Despite these limitations, the research confirmed the clinical importance of an unplanned reoperation in oral cancer surgery.

## Conclusion

This study suggests that tumor size, diabetes, N classification (N2), and pedicled flap reconstruction might be risk factors for UR after oral cancer radical surgery. UR was associated with worse long-term survival, and may be a novel prognostic factor for OC patients. These findings may help identify high-risk patients for UR, optimize surgical planning, strengthen perioperative management, and reduce the incidence of unplanned reoperations.

## Data Availability

The data are available from the corresponding author on reasonable request.

## Abbreviations

UR: Unplanned reoperation
OC: Oral cancer
BMI: Body mass index
